# What Are the Maternal Factors that Potentially Intervenes in the Nutritional Composition of Human Milk?

**DOI:** 10.3390/nu13051587

**Published:** 2021-05-10

**Authors:** Yasmin Amaral, Leila Silva, Fernanda Soares, Daniele Marano, Sylvia Nehab, Andrea Abranches, Ana Carolina Costa, Maria Elisabeth Moreira

**Affiliations:** 1 Unidade de Pesquisa Clínica, Instituto Nacional de Saúde da Mulher, da Criança e do Adolescente Fernandes Figueira (IFF), Fundação Oswaldo Cruz (Fiocruz), Rio de Janeiro 22250-020, Brazil; leila.silva@iff.fiocruz.br (L.S.); fevalente@gmail.com (F.S.); danielemarano@yahoo.com.br (D.M.); sylvia.nehab@gmail.com (S.N.); andreadunshee@gmail.com (A.A.); ana.costa@iff.fiocruz.br (A.C.C.); bebethiff@gmail.com (M.E.M.); 2 Programa de Pós-Graduação em Pesquisa Aplicada à Saúde da Criança e da Mulher, IFF, Fiocruz, Rio de Janeiro 22250-020, Brazil

**Keywords:** nutritional composition, human milk, associated factors, puerperal women

## Abstract

Background: To evaluate the potential factors associated with the nutritional composition of human milk of puerperal women. Methods: cross-sectional study, conducted between March 2016 and August 2017, with 107 women, selected in a Tertiary Health Care Tertiary Health Facility of the Unified Health System (SUS) in the Municipality of Rio de Janeiro. Data were collected two months after delivery. The dependent variable of the study was the nutritional composition of human milk. We divided the independent variables into hierarchical levels: distal (age, schooling, parity and pregestational nutritional status), intermediate (number of prenatal visits and gestational weight gain) and proximal (alcohol consumption, smoking, diabetes mellitus and hypertension). For data analysis, we applied the multiple linear regression, centered on the hierarchical model. Only the variables associated with the nutritional composition of breast milk remained in the final model at a 5% level of significance. Results: The nutritional composition of human milk yielded by women with pregestational overweight, smokers and hypertensive had higher amounts of lipids and energy. Conversely, women with gestational weight gain below the recommended had lower amounts of these components. Conclusion: The evaluation of factors associated with the nutritional composition of human milk is extremely important to assist post-partum care practices. In this study, we observed that lipid and energy contents were associated to pregestational nutritional status, gestational weight gain, smoking and hypertension.

## 1. Introduction

The human milk is a complex biological fluid with thousands of components of importance to the health, growth, development and immunity of the child [1]. It guarantees adequate nutrition for newborns and infants as a continuation of intrauterine nutrition [2]. Exclusively breastfeeding is recommended, as the only source of nutrients in the first six months of life. Thereafter, nutritious complementary foods will be introduced and breastfeeding continued up to the age of two years or beyond [3].

Lactose is the main carbohydrate of the human milk and provides about 45–50% of the total energy content [4]. Lipids, stratified into triglycerides (98%), phospholipids (1%) and sterols (0.5%) represent 30–50% of the calories. As for the proteins, 80% come from α-lactalbumin [5]. In addition to these macronutrients, human milk contains vitamins, minerals and numerous bioactive components, such as immunoglobulins, cytokines, hormones and oligosaccharides, among others [4].

The nutritional composition of human milk may be influenced by some maternal factors, such as the nutritional status of the pregnant woman [6], maternal age [7], maternal food intake [8], the lactation stage [9,10], circadian physiology, duration of breastfeeding (fraction emulsion, suspension or solution) [4], behavioral habits (smoking and alcohol) [11,12], maternal illnesses (hypertension and diabetes mellitus) [13] and the sex of the child [14], among others. Although studies discussing the potential elements associated with the nutritional composition of human milk cover a wide range of subjects, the results are not conclusive. There are no studies so far that evaluated simultaneously potential influences using a hierarchical model. The present study aims to evaluate potential factors associated with the nutritional composition of breast milk.

## 2. Materials and Methods

This is a cross-sectional study of puerperal women, two months after delivery, with data collected from March 2016 to August 2017. There were two eligibility criteria to enter the study: deliver in a tertiary public health unit in the city of Rio de Janeiro (RJ), gestational age equal to or greater than to 37 weeks and those followed in the same health unit for routine visits. Women with HIV+ diagnosis, previous exposure to Zika virus, congenital infection of the TORCH group (toxoplasmosis, rubella, cytomegalovirus and herpes), malformations, genetic syndromes, babies who received specialized care in neonatal intensive care unit and/or newborns who were unable to be breastfed at the time of the study did not participate in the study.

Trained interviewers collected the data. Once the postpartum women accepted to participate in the study, we applied structured questionnaires specifically designed for this research and pretested in a pilot study.

The dependent variable is the nutritional composition of human milk (carbohydrate, protein, lipid and energy value). The collection of human milk was performed using the Medela electric pump^®^ by a professional previously trained and wearing personal protection equipment (PPE—lab coat, cap, mask and disposable gloves), observing the same daily collection time frame (10:00–14:00 h) to prevent the variability in the nutritional composition of milk throughout the day. In cases of discomfort when using the pump, we shifted to manual milking. We collected milk samples, 10 mL of a total corresponding to total emptying of the nursing mother’s breast, stored in glass bottles with a capacity of 90 mL and subsequently frozen −20 °C.

We measured the macronutrients (carbohydrate, protein and lipids) and total energy with the spectrophotometry technique, through INFRARED ANALYSIS using Miris Human Milk Analyzer™ (Miris AB, Uppsala, Sweden) (already validated for human milk analysis) following good clinical laboratory practice [15,16].

The Miris Human Milk Analyzer™ was operated according to the manufacturer’s recommendations. Prior to analysis, a daily calibration check was performed using the calibration solution (Miris Calibration Control Kit™). These are standardized solutions with known concentrations of fat, protein and carbohydrates that correspond to those found in average human milk and the high end of the Miris Human Milk Analyzer™ measuring range.

For analysis, samples were thawed in a water bath (Miris Heater™) until the temperature reached 40 °C. Then, these samples were homogenized (1.5 s/1 mL) using a sonicator (Miris Sonicator™). The homogenized milk sample (1 mL) was injected into the flow cell and measured 60 s and macronutrient results were expressed in g/100 mL. To calculate the pregestational nutritional status, we used the cut-off points of body mass index (BMI) using pregestational weight and height recommended by the Institute of Medicine [17] the body mass index (BMI) calculated with pregestational weight and height. Women with low weight, BMI < 18.5 kg/m^2^, eutrophic with BMI ≥ 18.5–< 25 kg/m^2^, overweight with BMI > 25–< 30 kg/m^2^ and obese with BMI ≥ 30 kg/m^2^. We calculated the total gestational weight by subtracting the pregestational weight (baseline) from the weight of the last prenatal consultation. We copied the weight gained at the end of the pregnancy from the women’s pregnancy booklets. For the present study, we classified this variable into three categories (insufficient, adequate and excessive) also following the IOM guidelines [17]. We used data from the medical records to evaluate the presence of diabetes mellitus and hypertension before and during pregnancy.

**Figure 1:**
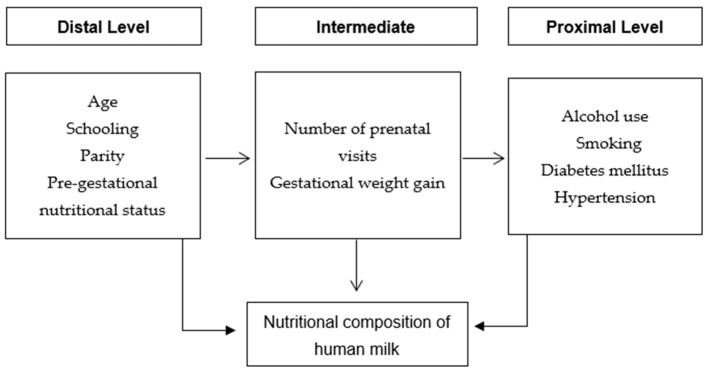
Hierarchical model of potential factors associated with the nutritional composition of human milk of puerperal women.

We used a hierarchical linear regression model to evaluate maternal determinants on the nutritional composition of human milk. We divided the factors potentially associated with the outcomes into three levels: distal (age, schooling, parity and pregestational nutritional status), intermediate (number of prenatal visits and gestational weight gain) and proximal (alcohol use, smoking, diabetes mellitus and hypertension, Figure 1). For each hierarchical group, we ran a bivariate analysis and the variables with *p*-value set below 0.20 to initial inclusion in the model. To evaluate the possible existence of multicollinearity we used the variance inflation factor (VIF). Backward elimination procedure was used to identify variables significantly related to the outcomes. In the final model, only the predictors associated with the nutritional composition of breast milk at a level of 5% remained. At each level, we controlled for all variables of the same level and for the higher level, but not for the lower-level variables. Thus, the estimate obtained at each hierarchical level concerns the effect of the variable already adjusted for possible confounding factors, but not for the possible mediating variables.

The study was approved by the Ethics Committee on Research with Human Beings of the National Institute of Women, Fernandes Figueira (CAAE 50773615.5.1001.5269) and is in accordance with the ethical principles of non-maleficence, beneficence, justice and autonomy, contained in resolution 466/12 of the National Health Council [18].

## 3. Results

We included 107 puerperal women in the study, 70.1% aged between 19 and 35 years, 62.6% multiparous and 38.3% declared themselves colored. Regarding behavioral habits, 10.3% consumed alcoholic beverages and 3.7% smoked (Table 1). Regarding clinical and obstetric characteristics, 31.1% had hypertension and 18.7% had diabetes mellitus before or during pregnancy. All postpartum women underwent prenatal care and the vast majority (95.1%) had more than six consultations. Regarding pregestational nutritional status, 46.3% were overweight. The percentage of women who gained weight adequately was 38.3% (Table 1).

Regarding the nutritional composition of human milk, in the second month after delivery, the median energy value was 55 Kcal/100 mL, carbohydrate 6.9 g/100 mL, proteins 0.9 g/100 mL and lipids 2.5 g/100 mL (Table 2).

This section may be divided by subheadings. It should provide a concise and precise description of the experimental results, their interpretation and the experimental conclusions that can be drawn.

In the bivariate analysis of the distal block, age (<19 years) and low weight pregestational nutritional status were associated with low human milk protein level, a significant reduction. Pregestational overweight was associated with increased energy and lipid values. In the intermediate block, gestational weight gain below the recommended was associated with a significant reduction in energy and lipid values. In the proximal block, smoking and systemic arterial hypertension were associated with increased energy and lipid values (Table 3).

The variables that remained in the final model were pregestational nutritional status, gestational weight gain, smoking and hypertension. It is noteworthy that overweight in the pregestational period, smoking and hypertension were associated with increased energy and lipid values in human milk. On the other hand, women with gestational weight gain below the recommended had a reduction in both lipids and energy values (Table 4; Supplementary Table S1).

## 4. Discussion

The application of the hierarchical model in the present investigation indicated that overweight in the pregestational period, gestational weight gain below the recommended, smoking and hypertension were associated with a change in the nutritional composition of human milk. There was an increase in the energy and lipid values among already overweight in the pregestational period. These results are similar to studies conducted by Fujimori et al. (2015) [19], Dritsakou et al. (2017) [10] and Hahn et al. (2018) [7].

Fujimori et al. (2015) [19] and Amaral et al. (2021) [20] observed that women were pregestational overweight had higher levels of fat and energy in the colostrum, with no differences in protein content between overweight and eutrophic women. Dritsakou et al. (2017) [10] also found higher levels of fat and energy in colostrum and transition milk of overweight women. Hahn et al. (2018) [7] conducted a cross-sectional study with 80 healthy Korean lactating women to evaluate the influence of body mass index and maternal age on the nutritional composition of human milk. They observed that women up to 30 years of age and overweight had a higher energy and lipid content than their older and slimmer counterparts. It is noteworthy that the influence of age and overweight in relation to the nutritional composition of human milk is not entirely clear so far. Among overweight women, a reduction of lactose synthesis can explain in part the higher content of fat in human milk. They have higher blood concentration of triglycerides and increased oxidative stress [13].

In 2020, a systematic review aims to identify the association between overweight and the nutritional composition of human milk. The study observed that eight papers showed that overweight increased the total concentration of lipids or glucose or macronutrient fractions, and only one study found no association between overweight and the nutritional composition of human milk. The authors recommend that women’s weight and height be evaluated in the pregestational visit to identify and monitor nutritional deviations, contributing to weight adequacy before pregnancy and assisting in the production of milk with adequate nutritional composition [6].

An increase in the prevalence of women who presented a weight gain curve above that recommended by the IOM (2009) [17] is already known. In the present study, in spite of the prevalence of inadequate weight gain being lower than in the other categories, these women showed a reduction in the amount of energy and lipids in human milk in the second month after delivery. Weight gain affects the maternal fat reserve and this reserve is likely to be a source for the lipid composition of milk [21]. We can speculate that these mothers with weight gain lower than recommended had also lower fat stocks, and consequently lower fat content in the milk.

Corroborating this result, Antonakou et al. (2013) [22] pointed out an association between body weight gain during pregnancy and the concentration of total fat in breast milk.

In the sample of the present study, only four women self-reported smoking. Even with this low prevalence in relation to this maternal behavior, the results of the multiple hierarchical analysis showed an increase in lipid and energy value in the milk of female smokers. However, Bachour et al. (2012) [11], in a study with 46 women, evaluated numerous factors associated with the modification of the nutritional composition of human milk and IgA concentrations, among which smoking stood out. The authors observed that the milk of smoking women contained less lipids and proteins. In addition, they observed that the concentration of IgA was lower among smokers. This disparity between the results of the aforementioned studies still needs elucidation.

Despite this issue, it is worth emphasizing the harmful effects that nicotine has on the baby and on the reduction of human milk production due to the inhibition of prolactin [12].

The literature has controversially pointed out a possible influence of maternal morbidities such as diabetes mellitus and arterial hypertension on the nutritional content of human milk [10,13,19,23]. Our results showed that maternal arterial hypertension increased the amount of energy and lipid value in human milk. However, the study conducted by Massmann et al. (2013) [9] observed that colostrum and mature milk of hypertensive mothers had higher levels of total protein, and that this increase reflected in the concentrations of antibodies and proteins of the immune system, increasing the levels of IgG, IgA and C3. The methodological differences between the studies may explain these disparate results such as the small sample size of the aforementioned study, where only 20 women (10 hypertensive and 10 not) were evaluated. Indeed, there are few studies on the association between hypertension and nutritional composition of milk. The results from a recent systematic review conducted by Amaral et al. (2019) [13], revealed that of the 14 articles selected, only one analyzed this question.

This study did not find a significant difference in the nutritional content of human milk of mothers with diabetes mellitus. However, Dritsakou et al. (2017) [10] identified higher levels of fat in the milk of diabetic women. This may be partly explained by the abnormal lipid metabolism in diabetics, which is marked by an elevation of lipoprotein lipase and lipolysis. It is worth noting that 50% of diabetics were diagnosed with overweight. However, the authors did not control any possible confounding factors. The systematic review carried out by Peila et al. (2020) [24] revealed, when examining the effect of diabetes mellitus in pregnancy on colostrum fatty acid composition, significantly higher concentrations of four essential omega-6 polyunsaturated fatty acids (γ-linolenic, eicosatrienoic, arachidonic and docosatetraenoic) in the colostrum of diabetic women, as compared to non-diabetic women [24]. Other studies show the potential influence of maternal food intake on the nutritional composition of human milk [25]. In this respect, the most studied macronutrient is lipid, and results are conflicting. Two 24-h recalls with one and two months after delivery is a methodology with well-known limitations. However, due to a lack of biological plausibility, we excluded this variable from the hierarchical model. The studies conducted by Iranpour et al. (2013) [26] and Zuraini et al. (2013) [27] demonstrated that maternal food intake during lactation did not modify the nutritional composition of human milk.

We acknowledge the limitations of our study. First, the analysis was restricted to a single moment of lactation. Although we chose to analyze only the mature milk, we collected an amount corresponding to total emptying of the nursing mother’s breast, minimizing the possible impacts of breastfeeding duration on the nutritional composition of human milk. Second, although there are other possible determinants associated with the nutritional composition of human milk, the present study did not evaluate them because they are not contained in the database of the larger project. Future studies will be planned to address this question. In addition, the main determinants associated with the composition of human milk described in the literature were analyzed in our hierarchical model. Lastly, as this is a cross-sectional study, we cannot establish causal relationships.

Our study has several strengths: we used controlled and consistent sampling protocols for collection and analysis of human milk; the present study offers in an unprecedented way the evaluation of potential maternal factors associated with the nutritional composition of human milk through a hierarchical model.

## 5. Conclusions

In the current study, it was possible to evaluate that lipid and energy were modified by pregestational overweight, gestational weight gain below the recommended, hypertension and smoking. Possibly, these factors did not change the amount of carbohydrate in human milk because this macronutrient has a close link to lactose synthesis and the amount of water drained into milk, aspects that lead to a smaller variation in its concentration. The evaluation of the possible factors associated with the nutritional composition of milk is of great relevance to establishment of services’ best practices and feedback efficient promotion of prenatal care. Therefore, the monitoring of women diagnosed with this aforementioned profile by the multidisciplinary team in the preconception and prenatal period is exceptionally relevant to minimize the impact of these factors on the nutritional content of human milk in order to provide an adequate growth and development of the infant.

## Figures and Tables

**Table 1:** Frequency of potential maternal factors associated with the nutritional composition of human milk of puerperal women attending a reference unit of the Unified Health System Network, 2016–2017.

Variables	*n*	%
Maternal age		
Up to 18 years	8	7.5
19–35 years	75	70.1
>35 years	24	22.4
Schooling (*n* = 106)		
Primary education	18	16.8
Secondary education	66	62.3
College+	22	20.8
Skin Color		
White	39	36.4
Colored	41	38.3
Black	22	20.6
Others	5	4.7
Parity		
Primipara	40	37.4
Multipara	67	62.6
Number of prenatal consultations (*n* = 102)		
<6	5	4.9
≥6	97	95.1
Alcohol consumption during pregnancy		
No	96	89.7
Yes	11	10.3
Smoking during pregnancy		
No	103	96.3
Yes	4	3.7
Drug consumption during pregnancy		
No	106	99.1
Yes	1	0.9
Pregestational nutritional status (*n* = 106)		
Low weight	5	4.7
Adequate weight	52	49.1
Overweight	34	32.1
Obese	15	14.2
Gestational weight gain ^1^ (*n* = 104)		
Below recommended	24	23.1
Adequate	41	38.3
Above recommended	39	37.5
Diabetes mellitus		
No	87	81.3
Yes	20	18.7
Hypertension (*n* = 106)		
No	73	68.9
Yes	33	31.1

^1^ Recommended by the Institute of Medicine (IOM. 2009).

**Table 2:** Energy and macronutrient content of human milk in the 2nd month after delivery of puerperal women treated in a reference unit of the Unified Health System Network, 2016–2017.

Human Milk Content	2nd Month after Delivery
Energy (kcal/100 mL)	55 (28–113)
Carbohydrates (g/100 mL)	6.9 (3.2–8.9)
Lipids (g/100 mL)	2.5 (0.5–8.6)
Protein (g/100 mL)	0.9 (0.3–9.0)

**Table 3:** Bivariate analysis of potential maternal factors associated with the nutritional composition of human milk in the 2nd month after delivery, 2016–2017.

	2nd Month after Delivery							
	Energy		Carbohydrates		Lipids		Protein	
Level 1 (Distal)	Effect	*p*-Value	Effect	*p*-Value	Effect	*p*-Value	Effect	*p*-Value
**Maternal Age in Years** <19	−1.58	0.80	0.17	0.59	−0.11	0.87	1.06	0.03
19–34	---		---		---		---	
>34	3.92	0.26	−0.11	0.54	0.40	0.29	−0.01	0.97
**Schooling**	0.36	0.63	0.02	0.68	0.04	0.62	0.08	0.20
**Parity**								
Primipara	---		---		---		---	
Multipara	−3.89	0.20	−0.10	0.50	−0.39	0.24	−0.37	0.13
**Nutritional status**								
Low weight	−5.28	0.42	−0.45	0.18	−0.32	0.66	1.10	0.04
Adequate weight	---		---		---		---	
Overweight	6.76	0.04	−0.18	0.28	0.73	0.04	−0.01	0.96
Obese	6.58	0.16	0.04	0.88	0.76	0.14	−0.18	0.64
**Level 2 (intermediate)**								
**Number of prenatal consultations**								
< 6	−4.56	0.55	0.20	0.59	−0.58	0.48	−0.19	0.76
≥ 6	---		---		---		---	
**Gestational weight gain**								
Below recommended	−9.62	0.01	0.24	0.21	−1.08	0.01	−0.26	0.42
Adequate	---		---		---		---	
Above recommended	−1.41	0.68	−0.26	0.12	−0.08	0.82	0.14	0.62
**Level 3 (proximal)**								
**Alcohol consumption**								
Yes	3.21	0.53	−0.26	0.30	0.39	0.48	−0.05	0.90
No	---		---		---		---	
**Smoking**								
Yes	26.30	0.01	−0.65	0.20	2.99	0.01	-0.12	0.88
No	---		---		---		---	
**Diabetes Mellitus**								
Yes	−1.07	0.78	0.12	0.52	-0.31	0.46	0.00	.1.00
No	---		---		---		---	
**Hypertension**								
Yes	8.22	0.01	−0.28	0.08	0.91	0.01	-0.03	0.91
No	---		---		---		---	

**Table 4:** Multiple analysis of potential maternal factors associated with the nutritional composition of human milk in the 2nd month after delivery, 2016–2017.

	2nd Month Post Delivery			
	Energy		Lipids	
	Estimated	*p*-Value	Estimated	*p*-Value
	(CI 95%)		(CI 95%)	
Block 1 (Distal)				
Pregestational nutritional status				
Low weight	−5.28	0.42	−0.32	0.65
	(−18.32; 7.77)		(−1.75; 1.10)	
Adequate	---	---	---	---
Overweight	6.76	0.04	0.73	0.04
	(0.30; 13.22)		(0.03; 1.44)	
Obese	6.58	0.16	0.76	0.14
	(−2.70; 15.86)		(−0.26; 1.77)	
Block 2 (Intermediate)				
Gestational weight gain				
Below recommendation	−8.47	0.03	−1.00	0.02
	(−16.08; −0.87)		(−1.82; −0.17)	
Adequate	---	---	---	---
Above recommendation	−2.33	0.49	−0.19	0.61
	(−9.00; 4.34)		(−0.91; 0.54)	
Block 3 (Proximal)				
Smoking				
Yes	23.67	0.02	2.65	0.02
	(3.76; 43.58)		(0.49; 4.81)	
No	---	---	---	---
Hypertension				
Yes	8.22	0.01	0.91	0.01
	(2.10; 14.34)		(0.24;1.57)	
No	---	---	---	---

## Supplementary Materials

The following are available online at https://www.mdpi.com/article/10.3390/nu13051587/s1, Table S1: Nutritional composition of human milk in the 2nd month after delivery according to maternal factors, 2016–2017.
