# Supplementary material for: What Are the Maternal Factors that Potentially Intervenes in the Nutritional Composition of Human Milk?
Source: Nutrients. 2021 May 10;13(5):1587. doi: 10.3390/nu13051587 (PMC8151127; doi:10.3390/nu13051587)
Supplement: Supplementary file 1 [file nutrients-13-01587-s001.zip › nutrients-1173649-supplementary.pdf]

nutrients

MDPI

Supplementary Table S1. Nutritional composition of human milk in the 2nd month after delivery according to maternal factors, 2016 – 2017.

	2nd month after delivery							
	Energy		Carbohydrates		Lipids		Protein	
**Level 1 (distal)**	Median	Range	Median	Range	Median	Range	Median	Range
**Maternal age in years**								
< 19	56.5	41 – 66	7.0	6.5 – 7.4	2.6	0.8 – 3.8	0.7	0.6 – 9.0
19-34	56.5	28 – 102	7.0	3.2 – 8.9	2.7	0.6 – 7.9	0.9	0.3 – 8.0
> 34	54.0	37 – 113	6.9	4.6 – 7.5	2.5	0.5 – 8.6	0.9	0.5 – 3.0
**Schooling**								
Primary education	59.5	39 – 82	7.1	4.6 – 8.9	3.1	0.6 – 5.5	0.8	0.3 – 9.0
Secondary education	54.0	37 – 100	6.9	3.2 – 7.7	2.5	0.5 – 7.7	0.9	0.4 – 3.0
College +	55.0	28 – 113	6.9	4.9 – 7.6	2.4	0.6 – 8.6	0.9	0.4 – 8.0
**Parity**								
Primipara	59.0	28 – 113	6.9	4.9 – 7.7	2.7	0.6 – 8.6	0.9	0.3 – 9.0
Multipara	53.5	37 – 100	7.0	3.2 – 8.9	2.3	0.5 – 7.7	0.9	0.4 – 2.3
**Nutritional status**								
Low weight	47.0	28 – 72	6.7	4.9 – 7.4	1.4	0.6 – 4.4	0.8	0.5 – 8.0
Adequate weight	53.0	37 – 100	7.1	3.2 – 7.7	2.3	0.5 – 7.7	0.9	0.4 – 9.0
Overweight	60.0	42 – 113	6.9	4.6 – 7.5	2.8	0.9 – 8.6	0.9	0.3 – 3.0
Obese	60.0	39 – 102	6.9	5.8 – 8.9	3.0	0.6 – 7.9	0.8	0.5 – 1.3
**Level 2 (intermediate)**								
**Number of pre-natal consultations**								
< 6	54.0	47 – 59	7.0	6.7 – 7.4	2.3	1.4 – 2.9	0.9	0.8 – 1.0
≥ 6	55.0	28 – 113	7.0	3.2 – 8.9	2.5	0.5 – 8.6	0.9	0.3 – 9.0
**Gestational weight gain**								
Below recommended	49.0	37 – 70	7.1	6.0 – 8.9	1.9	0.5 – 3.8	0.8	0.4 – 1.2
Adequate	58.5	39 – 113	6.9	5.3 – 7.7	2.8	0.7 – 8.6	0.9	0.4 – 8.0
Above recommended	57.0	28 – 102	6.9	3.2 – 7.6	2.8	0.6 – 7.9	0.9	0.3 – 9.0
**Level 3 (proximal)**								
**Alcohol consumption**								
Yes	59.0	49 – 77	6.8	4.6 – 7.4	2.5	1.9 – 5.0	0.9	0.5 – 2.3
No	54.5	28 – 113	7.0	3.2 – 8.9	2.6	0.5 – 8.6	0.9	0.3 – 9.0
**Smoking**								
Yes	83.5	65 - 102	6.2	5.8 – 6.6	5.7	3.5 – 7.9	1.0	0.8 – 1.1
No	55.0	28 - 113	7.0	3.2 – 8.9	2.5	0.5 – 8.6	0.9	0.3 – 9.0
**Diabetes Mellitus**								
Yes	54.0	40 – 82	7.2	4.6 – 7.5	2.4	0.6 – 5.1	0.9	0.4 – 3.0
No	55.0	28 – 113	6.9	3.2 – 8.9	2.6	0.5 – 8.6	0.8	0.3 – 9.0
**Hypertension**								
Yes	60.0	39 – 113	6.8	3.2 – 8.9	2.9	0.6 – 8.6	0.9	0.5 – 3.0
No	53.0	28 – 100	7.0	4.9 – 7.7	2.2	0.5 – 7.7	0.8	0.3 – 9.0
